# The value of initial cavitation to predict re-treatment with pulmonary tuberculosis

**DOI:** 10.1186/s40001-016-0214-0

**Published:** 2016-05-06

**Authors:** Qiusheng Huang, Yongmei Yin, Shougang Kuai, Yan Yan, Jun Liu, YingYing Zhang, Zhongbao Shan, Lan Gu, Hao Pei, Jun Wang

**Affiliations:** Center of clinical laboratory, The Fifth People’s Hospital of Wuxi, Affiliated to Jiangnan University, Wuxi, 214005 Jiangsu China; Department of Respiratory Medicine, The Second People’s Hospital of Huishan, Wuxi, 214005 Jiangsu China; Radiology department, The Fifth People’s Hospital of Wuxi, Affiliated to Jiangnan University, Wuxi, 214005 Jiangsu China

**Keywords:** Cavitation, Re-treatment, Pulmonary tuberculosis

## Abstract

**Objective:**

Pulmonary cavitation is the classic hallmark of pulmonary tuberculosis (PTB) and is the site of very high mycobacterial burden associated with antimycobacterial drug resistance and treatment failure. The objective of this study was to investigate the relationship between re-treatment PTB and initial pulmonary cavitation coordinated with other clinical factors.

**Methods:**

We conducted a case–control study of 291 newly diagnosed cases of pulmonary TB in The Infectious Hospital of Wuxi from Dec 2009 to Dec 2011 with complete follow-up information until December 31st of 2014. 68 patients were followed-up with PTB re-treatment; the rest of the PTB patients (*n* = 223) had completed anti-TB treatment, and cured without re-treatment were selected as controls.

**Results:**

The univariate analysis [hazard ratio (HR) 1.885, 95 % CI 1.170–3.035, *P* = 0.009] and the multivariable analysis (HR 2.242, 95 % CI 1.294–3.882, *P* = 0.004) demonstrated that the initial pulmonary cavitation was a prognostic predictor for TB re-treatment. Additionally, the re-treatment rates in PTB patients with cavitation and no-cavitation were 27.1 and 15.5 %, respectively, with significant difference (log-rank test; *P* = 0.010). Other factors, age of ≥60 and history of smoking, were also prognostic variables.

**Conclusion:**

Initial pulmonary cavitation of chest X-ray was a significant predictor for PTB re-treatment

## Background

According to Global tuberculosis report 2014, there were 5.7 million people had a new episode of tuberculosis (TB) and 0.4 million had already been diagnosed with TB but treatment was changed to a re-treatment regimen [1]. China and India are having the greatest burden of disease. India and China accounted for 22 and 15 % of total cases, respectively, in 2014 [1]. The burden of tuberculosis (TB) in South Africa is the third highest in the world. In the most Western European countries and United States, the majority of cases occur in foreign-born residents and recent immigrants from countries in which tuberculosis is endemic [2–4].

TB is presenting new challenges as a major health problem. The most concerning situation is the re-treatment of patients who are often exposed to conditions associated with future failures that are attributable to microbial resistance. The type of re-treatment TB included relapse, failure, treatment after default, or abandonment of treatment. The incidence of relapse TB in those who completed previous treatment can be 30 times higher than the incidence of TB in the general population [5]. Re-treatment of TB is associated with increased risk of drug resistance because of previous exposure to first-line anti-TB chemotherapy. In the recent global surveillance, it was estimated that 7.9 % of relapse cases were multidrug resistant TB for drug-resistant TB [6]. Re-treatment patients also have lower cure rate than incident TB and encounter more side effects during treatment with second-line drugs [7].

In this context, despite effective antimicrobial chemotherapy, re-treatment of tuberculosis (TB) after initial treatment remains a major challenge for TB control. Currently, more than 85 % initial patients were cured in China with an increase in coverage of directly observed treatment short-course (DOTS) therapy [8]. However, there still existed massive TB re-treatment patients in China due to the use of inadequate treatment regimens, the poor management of resistant cases, and the high transmission and mortality rates [8]. Moreover, with the large overall number of TB patients and the limited cure rate for re-treatment TB, the high overhead for re-treatment cases should not be ignored in China.

Effective predictive factors for TB re-treatment help to identify those at high risk of re-treatment disease and reduce the disease burden through early intervention. The pulmonary cavitation is the classic hallmark of TB and is the site of very high mycobacterial burden. Pulmonary cavitation is associated with antimycobacterial drug resistance [9] and treatment failure [10]. The presence of chest X-ray (CXR) cavitation is associated with a delayed therapeutic response. Patients with TB with pulmonary cavities are the principal source of disease transmission compared with those with non-cavitary disease [11–14]. However, there was still no direct evidence determining whether initial cavitation had the value to predict re-treatment with pulmonary tuberculosis. In this study, we conducted a case–control study to explore the value of initial cavitation to predict tuberculosis re-treatment.

## Methods

### Study population and ethics statement

**Fig. 1:**
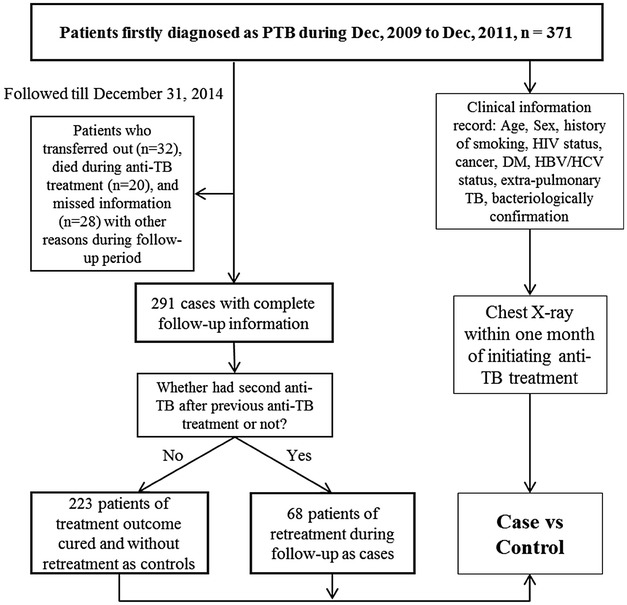
Flow diagram for the enrollment of study participants in the nested case–control study

We conducted a case–control study from 371 newly diagnosed cases of pulmonary TB in The Infectious Hospital of Wuxi from Dec 2009 to Dec 2011. A radiologist detected posterior–anterior CXRs of all newly diagnosed PTB patients for the presence or absence of cavities. A respiratory clinician reviewed all CXRs simultaneously. Then all of the newly diagnosed cases were admitted to our hospital to be hospitalized patients and received 2 months of isoniazid (H), rifampicin (R), pyrazinamide (Z), and ethambutol (E) during an intensive phase and 4 months of HR in the continuation phase. The duration of treatment was nearly 6 months. All information of patients was recorded in the clinical database of Infectious Hospital of Wuxi. We extracted the following information through medical chart review: age, sex, history of smoking, HIV status, cancer, DM, HBV/HCV status, coexisting extra-pulmonary TB, and bacteriologically confirmation. All patients were followed-up to December 31st of 2014. We excluded cases who transferred out (*n* = 32), died during anti-TB treatment (*n* = 20), and missed information (*n* = 28) with any other reasons during follow-up period. We adopted the WHO definition for TB treatment outcomes. After exclusion, finally, 291 patients with complete follow-up information were included in this study. Meanwhile, 68 patients were followed-up for TB re-treatment (Re-treatment was defined as patients who undergo second anti-TB treatment with active PTB after previous anti-TB treatment for one month or more). And, the rest of the no re-treatment PTB cases (*n* = 223) had completed anti-TB treatment and cured without re-treatment was selected as controls (Fig. 1).

This study was approved by the Institutional Ethics Committee of Infectious Hospital of Wuxi, Affiliated to Jiangnan University (No: WXIH2009-022), and was in compliance with the national legislation and the Declaration of Helsinki guidelines. Written patient consents were obtained according to the institutional guidelines.

### Statistical methods

Statistical analysis was conducted using SPSS version 17.0. Categorical variables were analyzed using Fisher exact tests and Chi-square tests, as appropriate, to identify significant differences across TB re-treatment cases and controls. Multivariable analysis was performed using Cox regression performed to determine the independent prognostic factors. Factors with a prognostic association in the univariate analysis were also entered into a multivariate Cox regression model. Results of the Cox regression modeling are presented as hazard ratios (HR) and associated 95 % confidence intervals (CI). The cumulative recurrence rate and curve during follow-up period was calculated using Kaplan–Meier analysis, and the log-rank test was utilized to examine the significance of the differences between different groups. *P* < 0.05 was considered as statistical difference for all analyses.

## Results

### Characteristics of the study participants

In the cohort study, we included all PTB patients from the study cohort during median follow-up period of 3.25 years (IQR 3.0–4.1 years) (*n* = 291). The median time from first treatment to second treatment of TB re-treatment patients was 1.25 years (IQR 0.9–1.9 years) (*n* = 68). Re-treatment was defined as patients who undergo second anti-TB treatment with active PTB after previous anti-TB treatment for one month or more. And, the rest of PTB cases who had completed anti-TB treatment and cured without re-treatment was selected as controls (*n* = 223).

The distribution of sex did not differ between cases and controls (*P* = 0.707) (Table 1). Compared to controls, cases were more likely to be patients with age ≥60 (*P* < 0.001), more likely to be smokers (*P* = 0.020), and more likely to present with initial cavitation (*P* < 0.001). The prevalence of DM, cancer, HBV/HCV, and extra-pulmonary lesion were higher among cases than controls (13.2 vs. 9.8 %, 8.8 vs. 3.6 %, 17.6 vs. 10.8 %, 23.5 vs. 18.8 %, respectively) without significance. Thirty-two (50.0 %) re-treatment cases had bacteriological evidence for active TB disease, and ninety-one (43.5 %) controls with bacteriological confirmation due to there were 2 cases and 14 controls missing information in bacteriologically test.

**Table 1:** Characteristics of the study participants

	Case (*n* = 68) *N* (%)	Control (*n* = 223) *N* (%)	*P* value
Cavitation on initial CXR	32 (47.1)	67 (30.0)	<0.001
Age (year)			
<60	22 (32.4)	101 (45.3)	<0.001
≥60	46 (67.6)	122 (54.7)	
Sex, male	50 (73.5)	169 (75.8)	0.707
History of smoking	42 (61.2)	102 (45.7)	0.020
Cancer	6 (8.8)	8 (3.6)	0.098
DM	9 (13.2)	22 (9.8)	0.441
HBV/HCV	12 (17.6)	24 (10.8)	0.145
Coexisting of extra-pulmonary lesion	16 (23.5)	42 (18.8)	0.153
Culture positive or/and smear-positive	32 (50.0)	91 (43.5)	0.364

Result of univariate analysis demonstrated that initial cavitation was a prognostic predictor for TB re-treatment [hazard ratio (HR) 1.885, 95 % CI 1.170–3.035, *P* = 0.009]. Other prognostic variables presented in Table 2 were age of ≥60 (HR 2.280, 95 % CI 1.371–3.790, *P* = 0.001) and history of smoking (HR 1.771, 95 % CI 1.086–2.888, *P* = 0.022). In the multivariable analysis, we included only 66 cases and 209 controls because of missing information in bacteriologically test. Initial cavitation of chest X-ray was also a prognostic predictor associated with re-treatment of TB (HR 2.242, 95 % CI 1.294–3.882, *P* = 0.004). Other prognostic factors, age of ≥60 (HR 2.044, 95 % CI 1.201–3.478, *P* = 0.008), history of smoking (HR 1.835, 95 % CI 1.012–3.321, *P* = 0.045), cancer (HR 2.831, 95 % CI 1.178–6.805, *P* = 0.020), HBV/HCV (HR 2.636, 95 % CI 1.343–5.172, *P* = 0.005), and coexisting of extra-pulmonary (HR 1.984, 95 % CI 1.094–3.598, *P* = 0.024) were also associated with TB re-treatment. However, gender, bacteriologically confirmation, and patients with DM were not confirmed to be prognostic factors with TB re-treatment in this study.

**Table 2:** Univariable and multivariable odds ratios for the associations between potential risk factors and TB re-treatment

	Unadjusted hazard ratio (95 % CI)	*P*	Adjusted hazard ratio (95 % CI)	*P*
Initial cavitation	1.885 (1.170–3.035)	0.009	2.242 (1.294–3.882)	0.004
Age (year)				
<60	Reference		Reference	
≥60	2.280 (1.371–3.790)	0.001	2.044 (1.201–3.478)	0.008
Sex, male	0.914 (0.533–1.567)	0.744	0.593 (0.311–1.130)	0.112
History of smoking	1.771 (1.086–2.888)	0.022	1.835 (1.012–3.321)	0.045
Cancer	2.269 (0.981–5.246)	0.055	2.831 (1.178–6.805)	0.020
DM	1.323 (0.656–2668)	0.434	1.864 (0.882–3.936)	0.103
HBV/HCV	1.514 (0.812–2.825)	0.192	2.636 (1.343–5.172)	0.005
Coexisting of extra-pulmonary	1.320 (0.754–2.313)	0.331	1.984 (1.094–3.598)	0.024
Culture positive or/and smear-positive	1.188 (0.733–1.926)	0.484	0.854 (0.494–1.477)	0.573

**Fig. 2:**
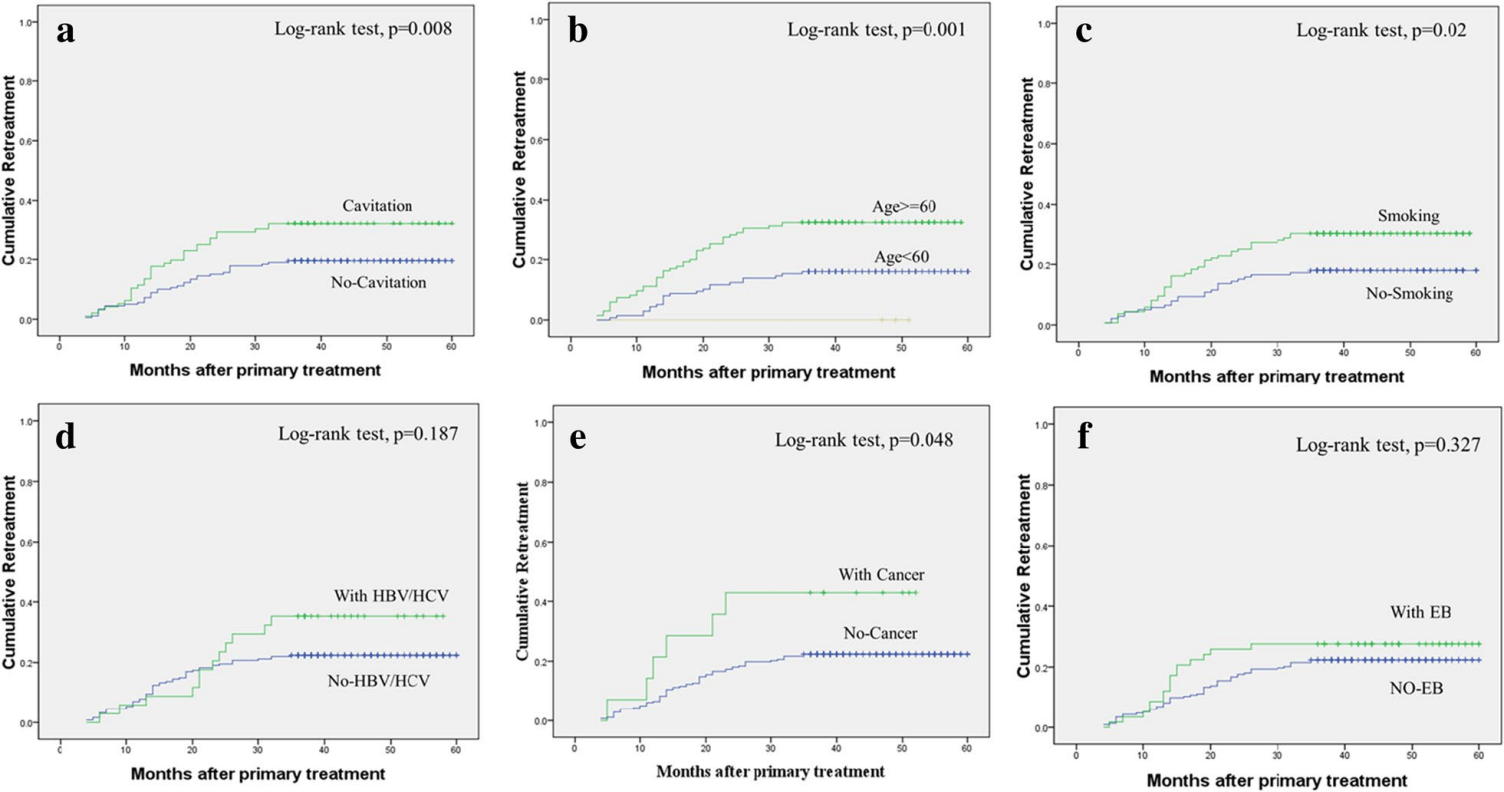
Cumulative re-treatment with months after primary treatment. **a** Cumulative re-treatment during follow-up period in APTB patients with cavitation and no-cavitation (Kaplan–Meier); **b**–**d** the different re-treatment rates in APTB patients with age of ≥60, history of smoking, and cancer compared to age of <60, no-smoking, and no-cancer (Kaplan–Meier); **e**, **f** Cumulative re-treatment during follow-up period in APTB patients with HBV/HCV and EB (extra-pulmonary) compared to no-HBV/HCV and no-EB (Kaplan–Meier)

The re-treatment rate increased with the follow-up time. The re-treatment rates in APTB patients with initial cavitation and no-cavitation are shown in Fig. 2; they were 32.3 and 18.8 %, respectively, with significant difference (log-rank test, *P* = 0.008). The different re-treatment rates in APTB patients with age of ≥60, history of smoking, and cancer compared to age of <60, no-smoking, and no-cancer were also significant (18.4 vs 15.3 %, log-rank test, *P* = 0.001; 29.2 vs 17.3 %, log-rank test, *P* = 0.02; 42.9 vs 32.4 %, log-rank test, *P* = 0.048, respectively). However, the different re-treatment rates in APTB patients with HBV/HCV and EB were without significant difference (log-rank test, *P* = 0.187 and log-rank test, *P* = 0.327, respectively).

## Discussion

Tuberculosis (TB) re-treatment still remains a major health problem despite an increase in coverage of directly observed treatment short-course (DOTS) therapy and adoption of passive case detection in China [8] due to perpetuate TB transmission and high rates of recurrent disease. Moreover, re-treatment of TB is associated with increased risk of drug resistance because of previous exposure to first-line anti-TB chemotherapy. Better understanding of the factors of TB re-treatment helps identify those at high risk of re-treatment disease and promises to reduce the disease burden through risk factor intervention.

Some investigators showed an association between anti-tuberculosis drug resistance [9] and treatment failure [10] with pulmonary cavitation; patients with TB with pulmonary cavities was the principal source of disease transmission compared with those with non-cavitary disease [11–14], and the presence of CXR cavitation is also linked with a delayed therapeutic response. In this study, we conducted a cohort study to explore the relationship between initial cavitation with tuberculosis re-treatment. The evidence indicated strongly that initial cavitation was a risk factor for PTB re-treatment and had the significant value to predict patients’ re-treatment with pulmonary tuberculosis. Moreover, the re-treatment rates in APTB patients with initial cavitation were higher than patients without cavitation during the follow-up time. It is the first report about the relationship between chest cavitation and PTB re-treatment as known.

Pulmonary cavitation is the classic hallmark of TB and is the site of very high mycobacterium tuberculosis (M.tb) burden. The high M.tb burden maybe one of causes for PTB re-treatment. M.tb infection triggers recruitment and infection of leukocytes and the activation of intercellular networks, which then result in damage, with tissue destruction [15]. Successful immune responses result in granulomas and curtailment of disease, while cavitation indicates a failing immune response [16]. Cavitation was associated with local neutrophilia and relative lymphopenia, whereas lymphocytosis and lower levels of granulocytes were detected from areas of pulmonary infiltrates and also from radiologically unaffected lobes [17]. The lymphopenia affected patients’ immune ability to prevent M.tb reactivation which could be inclined to disease re-treatment.

Other prognostic variables included age of ≥60 and history of smoking were also prognostic predictors associated with re-treatment of TB. Other independent risk factors for re-treatment included the presence of HBV/HCV and extra-pulmonary were associated with TB re-treatment in the multivariable analysis but not in the univariable analysis. However, gender, DM, cancer, and bacteriologically confirmation were not confirmed to be factors with TB re-treatment.

Our study also had limitations. The eligible patients represented a fraction of the patients diagnosed with active tuberculosis during the study period, raising a concern for a selection bias. Other risk factors for re-treatment in mainly new patients were described in other settings, such as health knowledge, distance to treatment center, and patients’ economic status; these could not however be assessed in this study [18, 19].

Except for these limitations above, the initial pulmonary cavitation in PTB patients was predictive for TB re-treatment. Additionally, the re-treatment rates in PTB patients with initial cavitation were higher than patients without cavitation. Other factors, especially patients with history of smoking, and age of ≥60 also had higher risk for re-treatment. Due to the large amount of TB patients and the limited cure rate for PTB re-treatment in China, the high overhead for PTB re-treatment cases should not be ignored. We recommend that chest X-ray monitoring are needed to confirm initial cavitation of PTB patients to control the risk of PTB re-treatment, an issue requiring early attention to avoid TB re-treatment.
